# Optical and Scintillation Properties of Record-Efficiency CdTe Nanoplatelets toward Radiation Detection Applications

**DOI:** 10.1021/acs.nanolett.2c02975

**Published:** 2022-11-04

**Authors:** Abhinav Anand, Matteo L. Zaffalon, Francesca Cova, Valerio Pinchetti, Ali Hossain Khan, Francesco Carulli, Rosaria Brescia, Francesco Meinardi, Iwan Moreels, Sergio Brovelli

**Affiliations:** † Dipartimento di Scienza dei Materiali, Università degli Studi di Milano-Bicocca, via R. Cozzi 55, 20125Milano, Italy; ‡ Department of Chemistry, Ghent University, 9000Ghent, Belgium; § Electron Microscopy Facility, Istituto Italiano di Tecnologia, Via Morego 30, 16163Genova, Italy

**Keywords:** CdTe nanoplatelets, GOST, exciton fine structure, scintillation

## Abstract

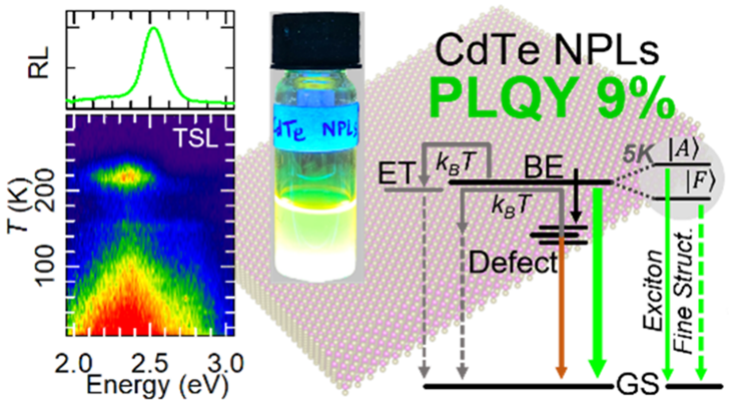

Colloidal CdTe nanoplatelets featuring a large absorption coefficient and ultrafast tunable luminescence coupled with heavy-metal-based composition present themselves as highly desirable candidates for radiation detection technologies. Historically, however, these nanoplatelets have suffered from poor emission efficiency, hindering progress in exploring their technological potential. Here, we report the synthesis of CdTe nanoplatelets possessing a record emission efficiency of 9%. This enables us to investigate their fundamental photophysics using ultrafast transient absorption, temperature-controlled photoluminescence, and radioluminescence measurements, elucidating the origins of exciton- and defect-related phenomena under both optical and ionizing excitation. For the first time in CdTe nanoplatelets, we report the cumulative effects of a giant oscillator strength transition and exciton fine structure. Simultaneously, thermally stimulated luminescence measurements reveal the presence of both shallow and deep trap states and allow us to disclose the trapping and detrapping dynamics and their influence on the scintillation properties.

Anisotropic two-dimensional nanoplatelets (NPLs) of cadmium chalcogenides (e.g CdS, CdSe, and CdTe) quantum-confined in one dimension (typically 1–2 nm thick with lateral dimensions up to tens of nanometers) have gained attention in the recent past due to their intriguing optoelectronic properties, leading to their potential application in a variety of emissive devices.^1−6^ Advancement in colloidal synthesis routes ensuring control over the thickness of NPLs with atomic precision have enabled the realization of sharp emission spectra (fwhm 8–10 nm), making them ideal candidates for high-color-purity displays.^2,7−10^ In addition, due to their suppressed Auger recombination^11,12^ and giant oscillator strength transitions (GOSTs),^13−16^ NPLs present themselves as some of the best candidates for low-threshold lasing.^17−20^ NPLs further exhibit large excitonic and biexcitonic binding energies stemming from a large electron–hole coupling due to the smaller dielectric constant of the surrounding medium.^21−25^ This results in a larger exciton–photon coupling strength, observed as faster radiative photoluminescence (PL) decay compared to 0D quantum dots or 1D nanorods.^1,26^ This has immense implications in their applicative potential in ultrafast photonic technologies, including fast scintillators^27,28^ for ionizing radiation detectors operating in a time-of-flight mode, which represent the essential component in high-resolution positron emission tomography scanners and calorimeters for high-luminosity colliders. This is further promoted by their composition based on heavy elements, such as cadmium and tellurium, whose high atomic number favors the interaction with high-energy photons and charged particles. Notably, ^116^Cd, ^130^Te, and ^82^Se are candidate isotopes for the study of neutrinoless double beta decay,^29,30^ a so far undetected, rare nuclear process whose observation would allow us to establish the neutrino mass, providing answers on the origin of the universe and unlocking unexplored scientific territories with unimaginable progress perspectives.

CdTe is characterized by a low band gap of 1.44 eV^31^ compared to its selenium- or sulfur-based counterparts (CdSe, ∼1.74 eV; CdS, ∼2.24 eV), which makes it an intriguing and tunable resource for application in photovoltaics^32^ and photodetection.^33^ These potential applications encouraged the development of procedures^34^ to synthesize colloidal spherical nanocrystals (NCs) of different sizes through organometallic^35^ and aqueous^36^ protocols. These methods were extended to make NCs of different shapes, resulting in colloidal CdTe nanorods,^37^ nanowires,^38^ nanotubes^39^ and tetrapods.^40,41^ However, the colloidal synthesis of CdTe NPLs and the studies conducted on them have lagged behind. Compared to their Cd chalcogenide counterparts, CdTe NPLs showcase widely separated and distinct light-hole (LH) and heavy-hole (HH) features, a narrow, tunable emission line width, and fast PL decay lifetimes among other interesting characteristics and can have a considerable impact in optoelectronics^42−45^ and photon management technologies.^46−48^ Coupling these with a large absorption coefficient and high atomic numbers (48 and 52 for Cd and Te, respectively), they present themselves as materials with enormous prospects in radiation detection.

However, CdTe NPLs are shown to suffer from low PL quantum yields (PLQYs) resulting from lattice defects which have pulled them down in the priority of research compared to CdSe NPLs. As a result, work on CdSe and CdS NPLs^1,49^ has been replicated only partially with CdTe,^1,50^ limiting its use in combination with other Cd chalcogenides such as CdSe and CdS in the form of core–shell,^51^ core–crown^45,52^ and core–barrier–crown heterostructures.^53^ To date, two**-**dimensional CdTe nanostructures have been reported in the form of zincblende structured NPLs,^1^ wurtzite structured nanoribbons^50^ and CdTe nanosheets.^54,55^ In 2013, Pedetti et al.^56^ optimized the synthesis protocol for the colloidal synthesis of CdTe NPLs with zincblende structure, yielding NPL populations of different thicknesses defined with atomic precision. Despite such advances, the emission PLQY of CdTe NPLs is still limited to ∼1%^1,55−58^ and no study has so far investigated their emission properties under ionizing radiation excitation.

In this work, we synthesize colloidal CdTe NPLs with the highest PL efficiency reported to date at PLQY = 9 ± 1%. We then study their photophysics by means of ultrafast transient absorption (TA) and temperature-dependent PL and performed radioluminescence (RL) and thermally stimulated luminescence (TSL) experiments, in an attempt to better understand the scintillation emission and competing (de)trapping phenomena. We find that the PLQY is limited by trapping in non-emissive defects whose suppression at low temperature boosts the excitonic emission by over 3-fold. In addition to this, a further radiative defect state is involved in the emission mechanism, resulting in a slow photo- and radioluminescence contribution that becomes fully radiative upon suppression of thermal quenching at cryogenic temperatures.

## Results and Discussion

The synthesis of 3.5 monolayered CdTe NPLs comprised of four layers of Cd atoms encompassing three alternating layers of Te atoms was inspired by the work by the Pedetti group.^56^ In our work, we use cadmium propionate (Cd(prop)_2_) as the Cd source and Te powder dissolved in tributylphosphine (TBP) as the source of Te atoms, as also recently attempted by Al-Shnani et al.^59^ and subsequently explored by Akhmetova et al.^60^ The effect of reactivities between anionic and cationic precursors and their carboxylic chain ligands has been shown to enhance the chemical yield and structural integrity of nanostructures.

Here, by using TBP-Te instead of the conventionally used TOP-Te, we observe the NPLs to be more orderly structured and smaller in lateral size, resulting in a much smaller scattering background in absorption spectra and HH-1S_e_ exciton transition at a slightly higher energy (2.51 eV) due to weak confinement effects along the lateral dimensions, as has been reported in CdSe NPLs. A lower lateral surface area would also mean a smaller probability of surface defects, resulting in a better PLQY. Moreover, using Cd(prop)_2_ ,which has one hydrocarbon longer chain than cadmium acetate (Cd(Ac)_2_) forces the reaction to be performed at a higher temperature (215 °C vs 170 °C), which leads to obtaining NPLs with higher crystal purity.^61−63^ Finally, to quench the reaction we used cadmium oleate (Cd(OA)_2_), a source of a Z-type ligand, instead of oleic acid that binds to the nanoplatelet (CdTe) surface and increases surface ligand concentration. This enhances the colloidal stability by preventing the formation of stacks and aggregation.^64^

**Figure 1:**
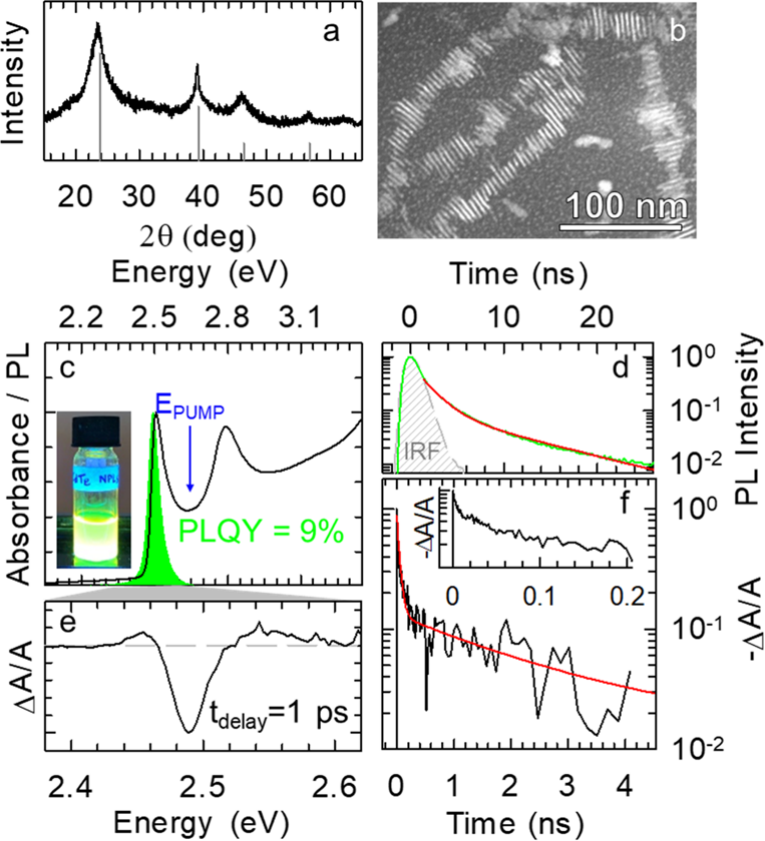
(a) X-ray diffraction pattern of CdTe NPLs showing a cubic zincblende structure (ICSD Collection no. 93942). (b) High-angle annular dark field-scanning transmission electron micrograph (HAADF-STEM) of the NPL ensemble. (c) Optical absorption and PL spectra (*E*_EXC_ = 3.54 eV, 300 K). Inset: photograph of a hexane NPL solution under UV light. (d) Normalized PL decay curve. The red line is the best fit with a double-exponential function; the measured instrument response function (IRF) is reported in gray. (e) Normalized TA spectrum collected at 1 ps after pumping at 2.69 eV. (f) Normalized TA kinetics for the HH-1S_e_ transition at 2.49 eV. The red line is the best fit with a triple-exponential decay function. The inset highlights the fast initial 10-fold drop in −Δ*A/A* signal.

**Figure 2:**
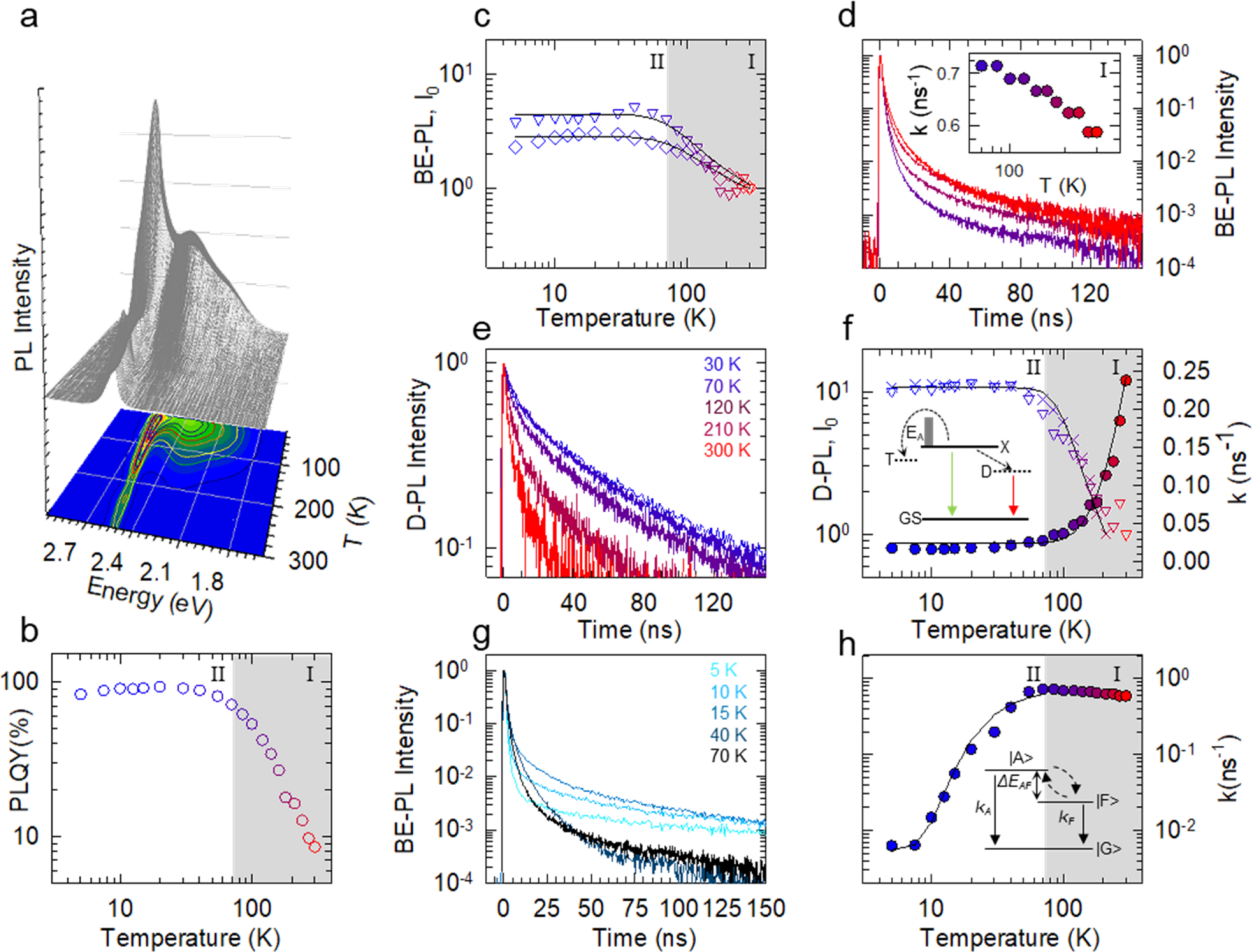
(a) PL spectra and (b) PLQY of CdTe NPLs as a function of temperature (300–5 K). (c) BE-PL intensities (diamonds) as extracted after deconvolution of the PL spectra in (a) and the respective zero-time intensities (triangles). The black lines are data fit with an Arrhenius function. (d) BE-PL decay curves vs*T* in range I. Inset: respective PL decay rates highlighting the influence of GOST. (e) Defect-PL decay curves as a function of temperature. (f) On the left *y* axis, temperature-dependent defect-related PL (D-PL, crosses) as extracted after deconvolution of PL spectra in (a) together with the result of the fitting procedure with the Arrhenius equation (black line), yielding an activation energy of 44 meV, and the zero-time defect related PL decay intensities (*I*_0_, empty triangles). On the right *y* axis, decay rates (*k*, solid circles) as extracted from defect-related PL decays fit using eq 2, yielding an activation energy of nonradiative decay of 48 meV. (g) PL decay curves at the BE showing accelerated PL profiles with decreasing temperature in range I as a consequence of GOST while being dominated by exciton fine structure effects (contrasting biexponential profiles) at lower temperatures (range II). (h) Temperature dependence of BE exciton decay rates (circles) in range I (300–70 K) showcasing the influence of GOST and in range II (*T* < 70 K), fit with eq 2 (black line) yielding a dark–bright splitting energy Δ*E*_AF_ of 5.4 meV. Inset: three-level model describing dark (|F⟩) and bright (|A⟩) exciton substates.

This synthesis protocol yields CdTe NPLs with dimensions 22 × 12 nm in zincblende crystal structure, as shown by the X-ray powder diffraction pattern and by the high-angle annular dark field-scanning transmission electron microscopy (HAADF-STEM) image reported in Figure 1a,b. As shown in Figure 1c, the NPLs are characterized by a sharp absorption feature at 2.51 eV corresponding to the first excitonic HH-1S_e_ transition followed by the LH-1S_e_ transition at 2.79 eV. The PL spectrum exhibits an ultrasharp peak with fwhm = 53 meV and no appreciable Stokes shift (<3 nm). The PL dynamics (Figure 1d) follows a double-exponential behavior with a fast decay component (τ_F_ = 2 ns) accounting for >85% of the PL intensity, followed by a longer-lived tail possibly originating from delayed emission from regenerated excitons from shallow defects states, as discussed in more detail in Figure 2. Crucially, the optimization of the synthesis protocol has resulted in a PLQY of 9 ± 1%. Despite this advancement, the still limited PLQY with respect to other cadmium chalcogenide NPLs indicates that exciton recombination in CdTe NPLs is still largely affected by nonradiative channels. To understand the origin of such losses, we turn to TA measurements, pumping the CdTe NPLs at 2.69 eV while operating at low pump fluences to avoid hot carrier losses and multiexcitonic effects. We start by looking at the early-time bleach spectrum shown in Figure 1e, having a feature at 2.49 eV due to filling of the first excitonic HH-1S_e_ state, consistent with the linear absorption measurements.^65−67^ An inspection of TA dynamics (Figure 1f) reveals a sub-nanosecond bleach recovery accounting for ∼90% of the total intensity followed by a slow tail matching the PL dynamics (τ ≈ 2 ns). We attribute the slow tail to the radiative decay of single excitons and the fast TA dynamics to charge trapping occurring in ∼200 ps. The close match between the PLQY value and the relative weight of the slow component of the TA dynamics (both ∼10%) strongly suggests that the dominant nonradiative loss is ultrafast charge trapping, which renders the majority of NPLs nonemissive. The same analysis conducted on standard CdTe NPLs featuring ∼1% PLQY is reported in Figure S2c (see also Methods in the Supporting Information), showing an analogous trend where ultrafast trapping accounts for >98% of optical losses. Overall, our TA results are consistent with the known inherent shallow traps resulting from surface defects in CdTe NCs and with time-resolved PL measurements reported in Figure 2, showing that most of the PL intensity loss occurs within the ∼1.5 ns time response of our time-correlated single-photon counting setup.^68−70^

To incisively study thermally activated processes affecting the PL efficiency and to investigate the fundamentals of exciton recombination, we performed cw and time-resolved PL measurements at low temperature. Figure 2a shows the PL spectral evolution of CdTe NPLs in the temperature range between 300 and 5 K. Decreasing the temperature leads to an enhancement in emission intensity associated with the band edge (BE-PL) accompanied by a broad sideband at ∼2.35 eV attributed to shallow emissive trap states (defect-PL). As shown in Figure 2b, cooling down the system leads to the growth of PLQY by over 10 times with respect to room temperature (RT), indicating that the total PLQY approaches unity. This result in itself might be misleading, since from Figure 2a it is observed that the PL has contributions from both BE and defect processes that can be seen to be more dominant at lower temperatures. To obtain better clarity of the two contributions, we decoupled the PL spectra using two Gaussian functions, as shown in Figure S4.

We start this part of the discussion with the BE-PL vs temperature (Figure 2c). Cooling the NPLs leads to a 3-fold enhancement in PL intensity up to 70 K (temperature range I) below which the intensity plateaus (temperature range II). This is accompanied by an increase in zero-time decay intensity, consistent with the suppression of the ultrafast carrier trapping observed in TA experiments. An Arrhenius fit (eq 1) yields an activation energy of 22 meV, consistent with the LO phonon energy in CdTe (21 meV),^71,72^ strongly suggesting that trapping is assisted by phonon coupling.
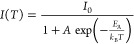
1Here, *I*(*T*) is the integrated PL intensity at a given temperature *T*, *I*_0_ is the integrated PL intensity approaching 0 K, *A* is a normalization constant, *E*_A_ is the activation energy, and *k*_B_ is the Boltzmann constant.

Interestingly, the PL intensification in temperature range I is accompanied by the acceleration of the PL decay (Figure 2d). Specifically, lowering the temperature leads to an evidently faster BE decay, as illustrated in the inset of Figure 2d, where we report the effective PL decay rates extracted as the inverse of the time at which the PL intensity drops by a factor of *e*. Fundamentally, such an acceleration of the PL kinetics is accompanied by an *increase* of the PLQY, indicating that in contrast to conventional emitters the behavior in temperature range I is not associated with larger nonradiative losses. In fact, the acceleration of the PL decay in CdTe NPLs is an implicative signature of GOST, as also observed in quantum wells^14^ and other colloidal NPL systems.^1^ GOST in colloidal NPLs arises due to the BE transition oscillator strength being concentrated to a single transition state with the lowest energy in *k*-space, resulting from strong, nonvarying confinement along the atomically precise thickness dimension in these NPLs. This leads to the exciton center of mass coherent motion extending all through the NPL, thus increasing its coherence volume.^14^ A greater coherence volume implies faster radiative transitions and thus higher decay rates. This phenomenon comes to the fore in systems deprived of phonon scattering and other nonradiative phenomena. Consequently, GOST can be understood and validated as an experimental observation of faster radiative decay rates accompanied by higher radiative PL intensities at lower temperatures.^1,14^ Moreover, the minor (<1%) long decay component is ascribed to phonon-mediated back transfer from the shallow trap states to the BE states, as also confirmed by its progressively lower intensity upon lowering the temperature.

Now, we look at the defect-PL intensity and kinetics as shown in Figure 2e,f. With a decrease in temperature, the defect PL exhibits a 10-fold intensification (Figure 2f) reaching saturation at ∼70 K. Concomitantly the PL decay rate, as extracted from Figure 2e, decreases by almost 1 order of magnitude (0.23 ns^–1^ vs 0.024 ns^–1^). This indicates that, on cooling the system, we may be exhausting the trapping channel into a nonradiative defect and also points to the fact that this defect is not the only one responsible for PL quenching. Concomitantly, looking at the defects, decreasing the temperature also results in an enhancement in zero-time decay intensity, along the same lines as defect-PL intensification, implying that trapping in this defect is not temperature activated. Also, since this trend is accompanied by increased zero-delay intensity of BE-PL, it also suggests that this defect is not the one mainly responsible for BE quenching, which is mostly affected by temperature-activated trapping in a nonemissive defect. In fact, on fitting the defect emission intensity with an Arrhenius plot, we obtain an activation barrier of 44 meV, corroborating the picture that quenching of BE and defect states occurs via unrelated channels.

Moreover, looking at the evolution of PL decay rate in Figure 2f, we can assign the decay rate in temperature range II to be the radiative recombination rate of trapped excitons *(*k^Tr^_RAD_). To quantitatively evaluate this, we perform a global fit of decay rates of trapped excitons (*k*^Tr^) and their respective temperature trends using the equation
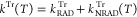
2where *k*_NRAD_^Tr^(T) is the nonradiative decay rate which can be fit using the standard displaced harmonic oscillator model^73^
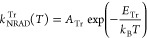
3The equation yields an activation energy of nonradiative decay of 48 meV, matching the value obtained for the corresponding PL intensities.

Based on the spectroscopic evidence, the PL kinetics can be explained by a possible model mechanism involving two defect states (Figure 2f). The state “T” is a nonemissive trap that captures excited carriers with the assistance of a LO phonon and renders the majority of NPLs nonemissive. This trap is coexistent with an emissive defect “D”, which has no activation barrier but is itself thermally quenched. It can be safely assumed that this defect would be different because the depth of localized states would couple with phonons differently from the way the excitonic state does.

Lowering the temperature further in range II, we move deeper into a purely radiative thermal environment marked by the invariant PLQY > 95% (Figure 2b,c). Cooling the system below 20 K results in a stark biexponential decay kinetics (Figure 2g) with an ultrafast component followed by a long-lived decay, over 2 orders of magnitude slower. The ultrafast component can be seen to become more assertive at lower temperatures, as shown for 5 K in Figure 2g and Figure S5. This behavior coupled with a constant PLQY presents a characteristic signature of exciton fine structure effects,^74,75^ well documented in CdTe QDs^74,76^ and CdSe NPLs^77,78^ but not currently for CdTe NPLs. The strong confinement of excitons, free to move across the two-dimensional plane of the NPL, gives rise to strong exchange interactions between uncorrelated electron–hole pairs, resulting in singlet–triplet exciton splitting. This effect is magnified at lower temperatures, since the exciton splitting energy exceeds the available thermal energy. Analogously to CdTe QDs^74,76^ and CdSe NPLs,^77,78^ the exciton ground state in CdTe NPLs is a 2-fold degenerate dark state |F⟩ with angular momentum projections ±2 separated from an optically bright state |A⟩ with angular momentum projection ±1 by a splitting energy Δ*E*_AF_. This is schematically depicted in the inset of Figure 2h. Here, the ultrafast decay component is a consequence of thermalization of excitons from the bright exciton state to the dark state while the much slower component is due to the recombination of the dark state. Δ*E*_AF_ can be extracted by assuming a Boltzmann distribution of excitons between these states by using the respective decay rates.
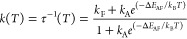
4The resultant Δ*E*_AF_ = 5.4 meV is in line with the dark–bright splitting energy observed in QDs of similar diameter^79^ and CdSe NPLs of similar thickness.^77^

The spectral analysis of the PL profiles vs *T* is reported in Figure S7.

**Figure 3:**
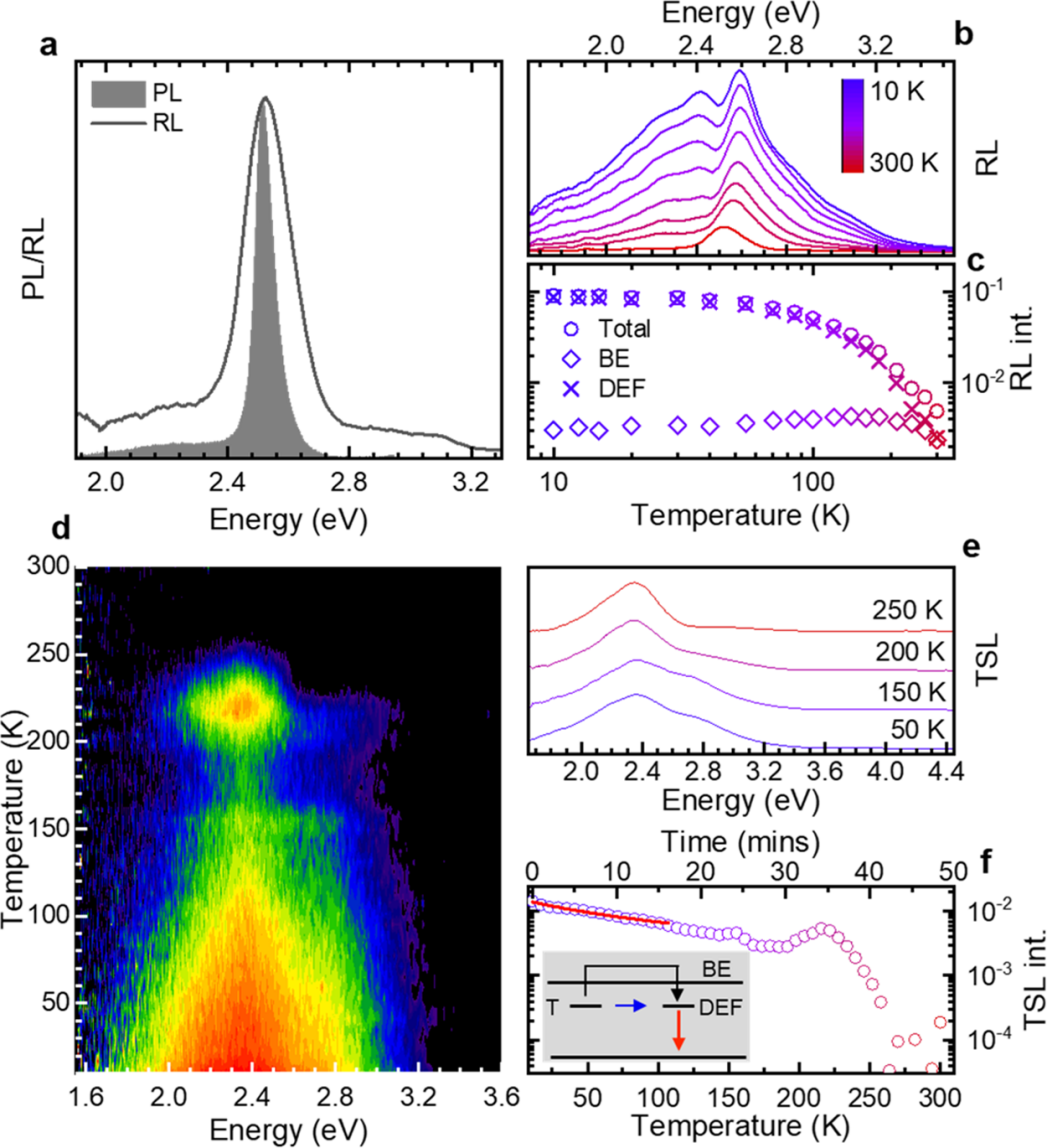
(a) RL and PL spectra at *T* = 300 K. (b) RL spectra vs temperature (10–300 K). (c) Temperature-dependent total RL (circles), BE-RL (diamonds), and defect-RL intensities (cross marks) as extracted from (b). (d) Contour plot of the spectrally resolved TSL intensity as a function of temperature. (e) TSL spectra and (f) glow curve at different temperatures extracted from (d). The red line in panel (f) is the fit in the low-temperature region to a power-law function. Inset: schematic depiction of the detrapping mechanisms.

In order to shed light on the surface defect states and on their influence on the scintillation ability of CdTe NPLs, we used RL spectroscopy as a function of temperature accompanied by TSL measurements below room temperature, which present a lucid idea of trapping and detrapping processes after charge generation by incident ionizing radiation. As exhibited in Figure 3a, the room-temperature RL is a single peak at 2.51 eV, similar to the PL spectrum, indicating that the lowest exciton recombination state is at 2.51 eV irrespective of whether the system is excited by X-rays or at 3.06 eV (note that the wider spectral width of the RL spectrum is mostly due to the lower resolution of the RL equipment, yet contributions to the spectral broadening due to multiexciton emission are also possible^27,28^). We estimated a radioluminescence yield of 500 ± 150 photons/MeV for CdTe NPLs dispersed in toluene solution with a concentration of 10^–4^ M, by comparison with the commercial liquid scintillator ULTIMA Gold AB (PerkinElmer Inc.) used as a reference and measured under the same experimental conditions. Figure 3b represents the RL spectra for 300 K < *T* < 10 K. As in the PL spectra (Figure 2a), upon decreasing the temperature from RT to 10 K, we observe a blue shift of around 70 meV in BE peak position and no measurable variation in spectral width. Decreasing the temperature below 210 K gives rise to a broad luminescence band at about 2.25–2.4 eV ascribed to the trapped excitons also responsible for the intragap low-*T* PL. The similarity between the PL and RL spectra as well as their temperature trends insinuate that such emissive defect states are intrinsic to the NPLs and are not created upon irradiation. Upon decreasing *T* (Figure 3c), we also observe a >10-fold enhancement and subsequent plateauing of the total RL intensity below 70 K. Along the same lines as the PL, the RL spectrum can be seen as a combinational result of BE and defect-related RL. Deconvolution of the RL spectra reveals that the BE-RL contribution is dominant at higher temperatures while the defect-related scintillation drives the RL intensities for *T* < 180 K.

To further disentangle the effect of temperature on the first steps of the scintillation process preceding the radiative recombination, specifically the charge generation, thermalization, and transport, we calculated the ratio between the integrated RL and PL intensity vs *T* (Figure S8) and obtain an almost flat profile for *T* < 200 K. This indicates that, below this temperature, scintillation acts efficiently in the generation and transport of charge carriers to the band edge and that the temperature dependence of both RL and PL is due to a thermal quenching of the radiative recombination. Interestingly, at higher temperatures, the RL/PL ratio increases by a factor of 2.5, indicating less-efficient trapping at unstable trap sites, as confirmed by TSL experiments that reveal the presence of stable trap states only below *T* = 220 K and their detrapping dynamics. The TSL contour acquired as a function of temperature (time) and energy (Figure 3d) shows a broad emission peaking at 2.35 eV (with a high-energy shoulder at 2.75 eV), consistent with luminescence from shallow defect states observed in PL and RL. By monitoring the TSL signal vs temperature (time), we further detect a sharp TSL peak at *T* = 220 K superimposed on a broad, monotonically decreasing signal. A more incisive context can be gathered by studying the so-called glow curve (Figure 3f) in terms of its kinetics. The monotonically decreasing trend more evident in the initial part of the curve is fitted by a power-law function of the form *I*(*t*) = *A*(*t* – *t*_0_)^−*p*^, yielding *p* = 1.1 ± 0.5, in agreement with direct a-thermal tunneling release of carriers to isoenergetic exciton states.^80^ In turn, the sharp TSL peak at T = 220 K indicates the onset of a thermally activated detrapping channel leading to the complete depletion of stable trap states at higher temperatures. The observed sharp decrease of the TSL intensity just after the peak at 220 K (Figure 3f) suggests that the same kind of trap is responsible for both a-thermal tunneling and the thermally assisted liberation of carriers, as depicted in the inset. Further detailed analysis of the origin and energetics of these trap states is beyond the scope of this work and warrants a separate specialized study.

In summary, we report the synthesis of colloidal CdTe NPLs with high PLQY, facilitating further optical and scintillation studies. The study underlines the effect of GOST at intermediate temperatures (100 K) and exciton fine structure effects for *T* < 50 K. TA and spectroscopic studies at a controlled temperature reveal a mechanism involving two defect states, a nonradiative trap and an emissive defect state competing with radiative exciton decay. TSL measurements confirm the presence of shallow and deep trap states and the signatures of detrapping dynamics enhancing the scintillation efficiency by a factor of 2.5 at higher temperatures.

## Supporting Information Available

Detailed description of the NPL synthesis and experimental methods and characterization data such as NPL size distribution, optical absorption, PL and TA spectra of control NPL samples, and PL spectral analysis as a function of temperature (PDF)
