# Supplementary material for: Optical and Scintillation Properties of Record-Efficiency CdTe Nanoplatelets toward Radiation Detection Applications
Source: Nano Lett. 2022 Nov 4;22(22):8900–7. doi: 10.1021/acs.nanolett.2c02975 (PMC9706671; doi:10.1021/acs.nanolett.2c02975)
Supplement: Supplementary file 1 — nl2c02975_si_001.pdf [file nl2c02975_si_001.pdf]

Supporting Information

Optical and scintillation properties of record efficiency CdTe nanoplatelets towards radiation detection applications

Abhinav Anand,† Matteo L. Zaffalon†, Francesca Cova,† Valerio Pinchetti,† Ali Hossain Khan,‡ Francesco Carulli,† Rosaria Brescia*, Francesco Meinardi,† Iwan Moreels,‡ * Sergio Brovelli†*

†Dipartimento di Scienza dei Materiali, Università degli Studi di Milano-Bicocca, via R. Cozzi 55, 20125 Milano, Italy

‡Department of Chemistry, Ghent University, 9000 Ghent, Belgium,

*Electron Microscopy Facility, Istituto Italiano di Tecnologia, Via Morego 30, 16163 Genova, Italy.

Methods:

Materials: Cadmium Oxide (CdO, ≥99.9%), Tellurium powder 200 mesh (Te, 99.8%), 1-Octadecene (ODE, ≥90%), Oleic acid (OA, ≥90%), Propionic acid (≥99%), Tributylphosphine solution (TBP, ≥93.5%) Hexane (≥97%), acetone (≥99%), ethanol (≥99%), and methanol (≥99%) were purchased from Sigma-Aldrich.

Precursors Synthesis:

Cadmium propionate ($Cd(prop)_2$): 2.59 g of CdO (20.1 mmol) and 25 mL of propionic acid were charged into a 50 mL flask. The mixture was heated at 70 °C for 1 hr in an inert environment. When the entire solid dissolved and the solution turned colourless, heating was stopped and brought to room temperature. The product was precipitated by addition of acetone. It was then filtered and washed with acetone, and dried under vacuum overnight.

Cadmium Oleate ($Cd(OA)_2$): 0.96 g of CdO (7.5 mmol) and 15 mL of Oleic acid were charged into a 25 mL. The mixture was heated at 200 °C for 1 hr in an inert environment. Once the solution turned colourless, the mixture was brought to 60 °C and degassed for 1 hr. After this, it was stored at room temperature and subsequently used in the reactions.

Tributylphosphine-Tellurium (TBP-Te) 1M: 255.2 mg Te powder (2 mmol) was dissolved in 2 mL TBP solution by constant stirring at 80 °C in an inert environment overnight.

CdTe 3.5 monolayered NPLs **synthesis:**

CdTe 3.5 monolayered NPLs: A 50 mL three neck flask was charged with 130 mg $Cd(prop)_2$ (mmol), 160mL OA (mmol) and 15mL ODE and the mixture was degassed at room temperature for 30 minutes and then at 95 °C for 60 mins. The Cd precursor is then dissolved in the non-coordinating solvent under a constant nitrogen flux and the temperature is set to 215 °C. When this temperature is reached, 100uL TBP-Te (1M) solution dispersed in 1 mL ODE was rapidly injected. This was accompanied by an immediate color change to yellow and then to yellowish orange. The reaction was allowed to continue for 30 mins after which the heating mantle was taken off and the flask was cooled down using compressed air to prevent undesirable formation of thinner NPLs. At about 120 °C, 1mL $Cd(OA)_2$, already heated to 100 °C was injected and the reaction was quenched. Once at room temperature, the NPLs were washed and precipitated with hexane and ethanol (1.5:1 ratio) using a centrifuge at 6500 rpm for 10 mins. This purification cycle was repeated one more time to get rid of unreacted precursors and excess ligands.

Powder X-Ray diffraction: Powder XRD patterns were acquired in Bragg–Brentano geometry with Cu Kα radiation (Panalytical X'Pert Pro powder diffractometer)

Transmission Electron Microscopy (TEM):

High-angle annular dark field-scanning TEM (HAADF-STEM) images were acquired using an image-Cs-corrected JEOL JEM-2200FS S/TEM, operated at 200 kV, at the Electron Microscopy Facility of Istituto Italiano di Tecnologia. For these analyses, the colloidal sample was drop-cast onto a Cu grid coated with an ultrathin amorphous carbon film.

Spectroscopic studies: Absorption spectra of NCs in solution were measured with a Cary 50 UV–vis spectrophotometer. Steady-state PL measurements were performed by exciting samples at 3.06 eV with ps-pulsed diode lasers. The emitted light was dispersed with a spectrometer and detected with a charge-coupled device. Transient PL measurements were carried out using ≈70 ps pulses at 3.06 eV from a pulsed diode laser (Picoquant

LDH-P series). The emitted light was collected with a phototube coupled to time-correlated single-photon counting unit (time resolution ≈ 600 ps). Temperature-dependent PL and time-resolved PL measurements were carried out on NC thin films drop-casted on quartz substrates and mounted inside a cryostat with optical access. PLQY measurements were carried out in an integrating sphere by exciting the NPLs sample with a continuous wave diode laser at 3.1 eV. The emitted light was dispersed with a spectrometer and detected with a charge-coupled device.

Ultra-fast transient absorption spectroscopy: these measurements are performed on the Helios TA spectrometer from Ultrafast Systems. The laser source was a 10 W Hyperion amplified laser which provided ~260 fs pulses at 1030 nm with a repetition rate of 2.14 kHz. The excitation pulses at 2.69 eV were obtained by the APOLLO-Y Optical Parametric Amplifier from the same vendor. The probe beam was a white light supercontinuum. The pump fluence on the sample was ~80μJ cm^{-2}. The CdTe NPLs were dispersed in toluene and kept under vigorous stirring during the TA measurements.

Radio-Luminescence: Samples were excited by unfiltered X-ray irradiation using a Philips PW2274 X-ray tube with a tungsten target, equipped with a beryllium window, and operated at 20 kV and 20 mA. At this operating voltage, X-rays are produced by the Bremsstrahlung mechanism due to the impact of electrons generated through a thermionic effect and accelerated onto the tungsten target. The RL was collected using a custom apparatus featuring a liquid nitrogen-cooled, back-illuminated, and UV-enhanced charge-coupled device (CCD) detector (Jobin-Yvon Symphony II) coupled to a monochromator (Jobin-Yvon Triax 180) with a 100 lines/mm grating. Cryogenic RL measurements are performed in the 10-300 K interval.

Thermally stimulated luminescence (TSL): Wavelength-resolved TSL at cryogenic temperatures is carried out by using the same detection system as for RL measurements. Cryogenic TSL measurements are performed in the 10−320 K interval, with a linear heating rate of 0.1 K/s, after continuous X-ray irradiation up to 5 Gy. The dose values were evaluated in air with an ionization chamber. The so-called glow curve represents the integrated TSL intensity as a function of both temperature and time, since in a TSL experiment temperature and time are linearly correlated.

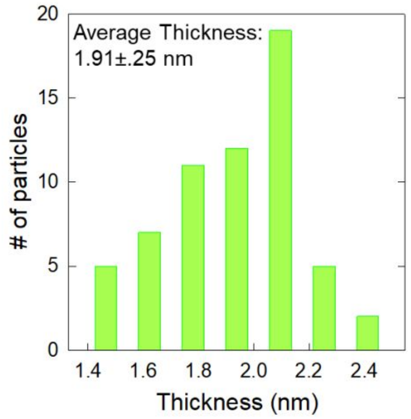

Figure S1: CdTe NLPs thickness distribution and average thickness extracted from HAADF-STEM images for 60 nanoparticles. The average thickness matches the expected thickness of CdTe NPLs (3.5 ML, ICSD 93942) featuring an adsorption edge at 2.49 eV.

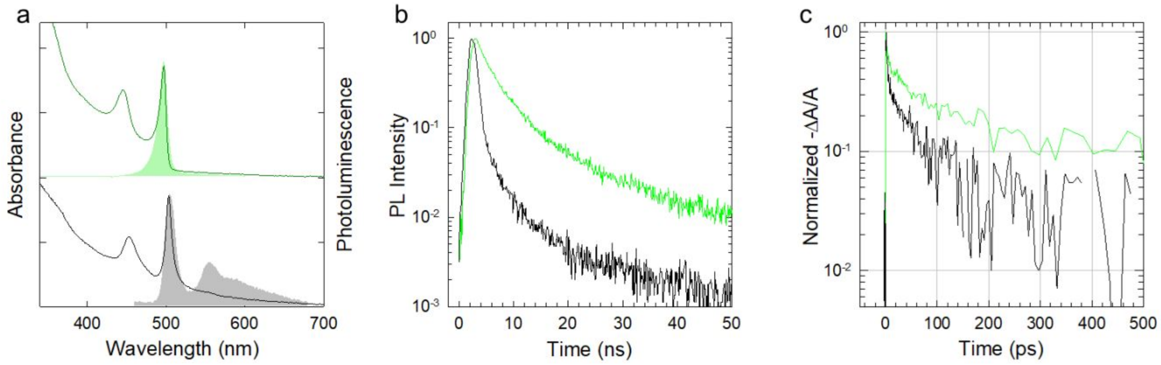

Figure S2. a) Absorption (line) and emission (shaded area) spectra of our highly efficient CdTe NPLs (green) and standard CdTe (black) NPLs. b) PL decay curves and c) Normalized transient absorption kinetics for the HH-1Se transition of highly efficient CdTe NPLs and standard CdTe NPLs. The same color code applies to all panels.

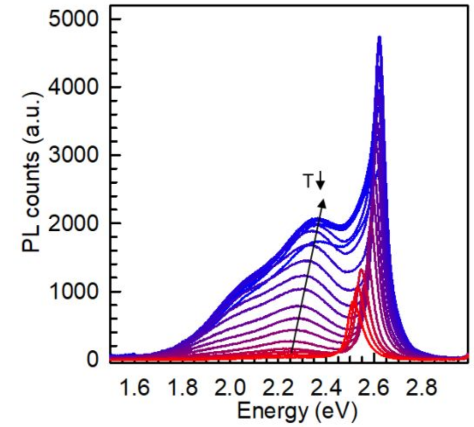

Figure S3: Non-normalized PL spectra at various Temperature points

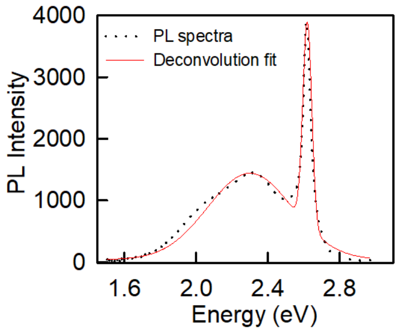

Figure S4: Gaussian deconvolution of PL spectra at in temperature range II

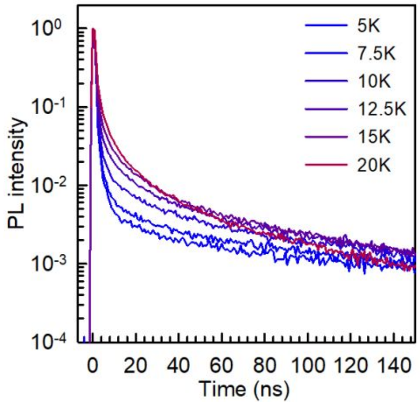

Figure S5: Bi-exponential evolution of PL decay curves at low Temperatures. (5-20 K)

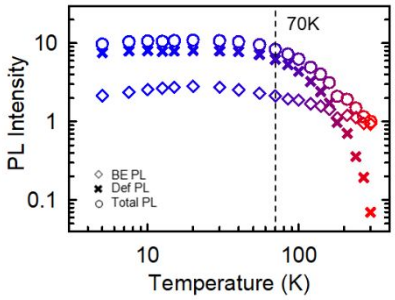

Figure S6: Integrated PL intensity of BE-PL and Trap emission contributions.

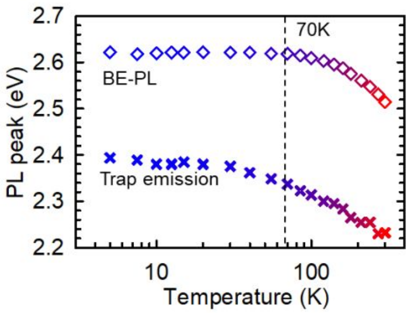

Figure S7: Peak intensity evolution of BE-PL and Trap emission contributions. Cooling down the system results in both BE and defect-PL peak shifting to higher energies. Between 300K and 70K, BE peak energy blue shifts by about 0.104 eV (2.512 eV at 300 K to 2.618 eV at 70 K) with no appreciable change in its spectral width (FWHM~ 70 meV) while for the defects related emission, the shift in peak energy is around 0.105 eV. This can suitably be ascribed to the contraction of the lattice constant in CdTe unit cells at cryogenic temperatures as also reported previously in spherically confined nanocrystals.

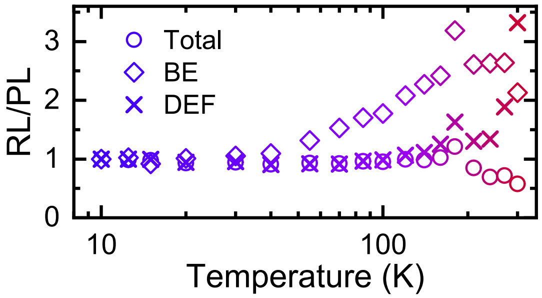

Figure S8: RL as a function of temperature normalized for its respective PL, for total intensity (circles), BE (diamonds), and defect-related emission (cross-marks).
